# When a Transient Ischemic Attack Is Not a Transient Ischemic Attack: Super-refractory Status Epilepticus in GAD65 Antibody-Associated Autoimmune Encephalitis

**DOI:** 10.7759/cureus.112292

**Published:** 2026-07-08

**Authors:** Santiago Luna-Alcala, Daniel L Olguín-Ramírez, Yaima C Pino-Peña, Miguel A Celis-Lopez, Jorge O García-Méndez, Victor M De la Puente-Diaz de Leon, Miguel E Rojas-Evaristo, Cinthia Rodríguez Juárez, Andrea S Pineda Venegas

**Affiliations:** 1 Department of Internal Medicine, Medica Sur Clinic and Foundation, Mexico City, MEX; 2 Department of Neurology and Neurosurgery, Medica Sur Clinic and Foundation, Mexico City, MEX; 3 Department of Infectology, Medica Sur Clinic and Foundation, Mexico City, MEX; 4 Department of Critical Care Medicine, Medica Sur Clinic and Foundation, Mexico City, MEX; 5 Department of Neurophysiology, Medica Sur Clinic and Foundation, Mexico City, MEX

**Keywords:** anti-gad65 autoimmune encephalitis, case report, cerebrospinal fluid antibody testing, super-refractory status epilepticus, transient ischemic attack mimic

## Abstract

A 68-year-old woman with no prior medical history was diagnosed with probable glutamic acid decarboxylase 65 (GAD65) antibody-associated autoimmune encephalitis, which initially mimicked a transient ischemic attack (TIA) before progressing to super-refractory status epilepticus. She initially experienced a transient episode of right-sided weakness and dysarthria, leading to a presumed vascular diagnosis. Eight days later, she returned with right central facial palsy and conduction aphasia, which rapidly progressed to global aphasia and was followed by fever and seizures, prompting neurological reevaluation. Electroencephalography (EEG) demonstrated encephalopathic activity with epileptiform discharges, while cerebrospinal fluid (CSF) analysis revealed isolated protein elevation without pleocytosis. Extensive investigations for infectious, neoplastic, neurodegenerative, and systemic autoimmune causes were unrevealing. Brain fluorodeoxyglucose-PET/CT (FDG PET/CT) demonstrated diffuse cortical hypermetabolism, a finding compatible with autoimmune encephalitis but one that may also have been influenced by ongoing or recent ictal activity.

Given the high suspicion for autoimmune status epilepticus, empiric treatment with methylprednisolone and intravenous immunoglobulin (IVIG) was initiated before the results of CSF autoimmune antibody testing became available. The patient subsequently demonstrated progressive clinical and electrographic improvement. After a prolonged ICU stay, she recovered with minimal disability. Follow-up serum testing revealed low-titer anti-GAD65 antibodies, which supported the diagnosis despite negative CSF autoimmune antibody testing. This report highlights the diagnostic challenges of GAD65 antibody-associated encephalitis presenting with cerebrovascular-like episodes, the potential discordance between serum and CSF antibody testing, and the importance of early empiric immunotherapy in suspected autoimmune encephalitis, even when initial diagnostic studies remain inconclusive.

## Introduction

Anti-glutamic acid decarboxylase 65 (anti-GAD65) encephalitis is a rare autoimmune syndrome caused by antibodies targeting GAD65, the rate-limiting enzyme involved in gamma-aminobutyric acid (GABA) synthesis within inhibitory synaptic terminals [1,2]. Its main neurological phenotypes include limbic encephalitis with temporal lobe epilepsy, stiff-person syndrome spectrum disorders, and cerebellar ataxia [1,3,4]. These clinical manifestations typically develop in a subacute or chronic manner. The case we present here is atypical in two respects: it began with recurrent, transient ischemic attack (TIA)-like episodes, and it evolved into super-refractory status epilepticus rather than the more common chronic epilepsy or limbic phenotype.

Seizures are a dominant feature of anti-GAD65 limbic encephalitis and are reported in most published cohorts [5]. Unlike surface-antigen antibodies, anti-GAD65 targets an intracellular epitope, and diagnostic evaluation should include both serum and cerebrospinal fluid (CSF) testing, as results may be discordant. In this case, the diagnosis was supported by serum anti-GAD65 positivity despite negative CSF testing, a previously described pattern that underscores the importance of dual compartment assessment [6]. Because low-titer serum positivity is less specific than high-titer or intrathecally synthesized antibodies, GAD65 serology must be interpreted within the clinical context and integrated with other findings rather than being considered standalone proof [5].

Cerebrovascular-like presentations preceding encephalitis features are exceedingly rare [7]. Super-refractory status epilepticus, defined as status epilepticus persisting or recurring despite ≥ 24 hours of anesthetic therapy, has been described only in limited case reports and small series involving anti-GAD65 antibodies [8]. The coexistence of these atypical features remains poorly characterized and poses a major diagnostic challenge. We report a case highlighting the diagnostic complexity of an initially CSF-negative presentation with subsequent low-titer serum positivity, the value of dual-compartment assessment, and the role of early empiric immunotherapy [9].

## Case presentation

Patient information

A 68-year-old woman with no medical history, chronic medications, or family history of autoimmune or neurological disease presented with two transient focal neurological episodes over eight days. Ten days before symptom onset, she had returned from a 10-day trip to Japan and developed a brief, self-limited febrile illness characterized by generalized weakness, dizziness, headache, and low-grade fever, which was treated empirically with a nonspecific antibiotic.

Clinical findings

First Visit 

The patient presented to the emergency department after a single transient episode of right-sided weakness involving the upper and lower limbs and the lower face, accompanied by dysarthria. Symptoms lasted approximately 20 minutes and resolved spontaneously before arrival. On admission, she was alert and fully oriented, with a Glasgow Coma Scale score of 15/15 and a normal neurological examination. Non-contrast head CT revealed no acute intracranial abnormalities, hemorrhage, or early ischemic changes. Given the focal and transient symptoms, a vascular workup was performed. Carotid duplex ultrasonography revealed a small echolucent atheromatous plaque in the left carotid bulb, causing a non-hemodynamically significant stenosis.

Transthoracic echocardiography showed no structural or functional cardiac disease, intracardiac thrombus, or right-to-left shunt. The ABCD2 score (Age, Blood pressure, Clinical features, Duration of symptoms, Diabetes; range 0 to 7) was 4, indicating a moderate short-term stroke risk (approximately 4% within two days) [10]. Following a standard cerebrovascular protocol (including CT, carotid duplex ultrasonography, transthoracic echocardiography, and ABCD2 risk stratification) and with a working diagnosis of TIA, she was discharged on a short course of dual antiplatelet therapy (consistent with guideline-based management of high-risk TIA and current secondary prevention guidelines) and scheduled for outpatient neurology follow-up [11,12,13].

*Second Visit* 

Eight days after the initial event, the patient returned with new focal neurological deficits. Examination showed right central facial palsy and conduction aphasia (NIHSS 2; the National Institutes of Health Stroke Scale ranges from 0 to 42, and a score of 2 indicates a minor deficit) [14]. Brain MRI was performed to exclude acute ischemia. Diffusion-weighted imaging (DWI) showed no restricted diffusion, and fluid-attenuated inversion recovery (FLAIR) sequences revealed only mild nonspecific white matter hyperintensities consistent with a Fazekas score of 1, without acute parenchymal lesions, mass effect, or abnormal enhancement [15].

**Figure 1:**
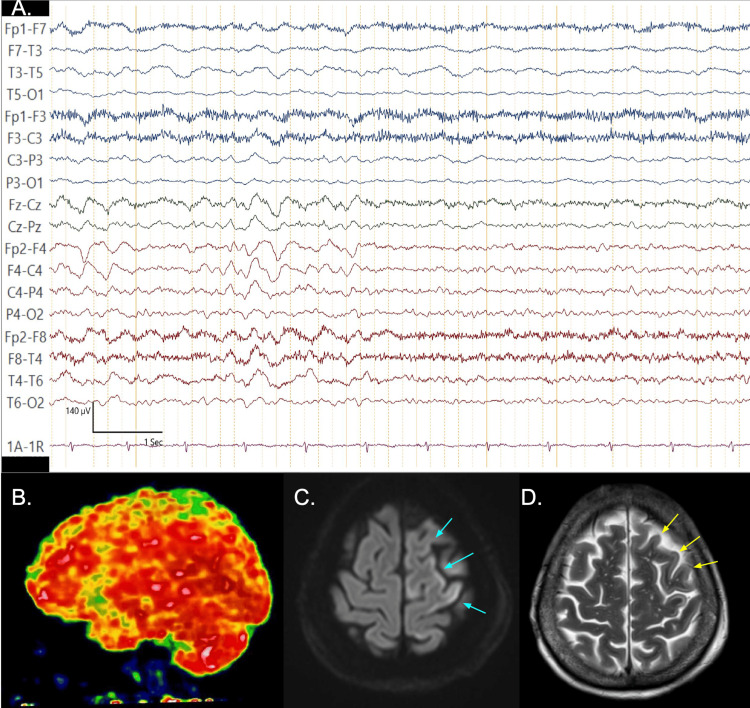
Electroencephalographic and multimodal neuroimaging features during the acute encephalopathic phase (A) Initial EEG obtained on admission demonstrated bilateral polymorphic slowing with marked interhemispheric asymmetry, characterized by lower-voltage activity over the left hemisphere, consistent with diffuse cerebral dysfunction and encephalopathy. (B) Initial brain 18F-FDG PET/CT showed diffuse cortical hypermetabolism (red areas on the color scale, indicating the highest FDG uptake), a supportive but nonspecific finding compatible with autoimmune encephalitis and/or sustained peri-ictal metabolic activity in prolonged status epilepticus. (C) Diffusion-weighted MRI demonstrated focal cortical diffusion restriction compatible with cytotoxic edema (blue arrows). (D) T2-weighted MRI showed cortical and subcortical hyperintensities with a nonvascular distribution, consistent with vasogenic edema related to sustained epileptic activity (yellow arrows) EEG: electroencephalography; 18F-FDG PET/CT: 18F-fluorodeoxyglucose positron emission tomography/computed tomography; MRI: magnetic resonance imaging

Over the following hours, conduction aphasia progressed to global aphasia, and left patellar clonus appeared, suggesting bilateral cortical involvement rather than a single vascular territory. Urgent electroencephalography (EEG) showed diffuse encephalopathic activity with superimposed frontotemporal sharp waves, inconsistent with a purely ischemic process and suggestive of an encephalopathic syndrome (Figure 1A). Lumbar puncture was performed for CSF analysis; during the procedure, she developed two generalized tonic-clonic seizures lasting one and five minutes, marking progression to status epilepticus. She also became febrile, with a peak temperature of 38.2 °C. She was intubated for airway protection and admitted to the ICU. Continuous video-EEG monitoring was started, and seizure control was achieved only after anesthetic titration to a burst-suppression pattern.

Diagnostic assessment

Given the rapid progression from transient focal deficits to super-refractory status epilepticus with fever, a broad workup was performed for infectious, neoplastic, systemic autoimmune, neurodegenerative, and neuronal antibody-mediated etiologies, reflecting the limited specificity of the initial clinical and EEG findings.

Infectious Workup

Central nervous system (CNS) infection was prioritized given the febrile encephalopathic presentation. CSF bacterial, fungal, and mycobacterial cultures were sterile; the FilmArray meningitis/encephalitis multiplex PCR was negative; and CSF adenosine deaminase was normal. Additional testing for HTLV-1/2, West Nile virus IgM, Borrelia burgdorferi, Rickettsia spp., enteroviral PCR, rubella IgM, measles, and HIV was negative. Metagenomic next-generation sequencing, which can identify low-abundance or fastidious pathogens beyond the reach of culture and targeted PCR, was not available at our centre. Despite recent travel to Japan and the preceding self-limiting febrile illness, the absence of infectious markers strongly argued against infectious encephalitis.

Systemic Autoimmune and Neurodegenerative Workup

Serum and CSF testing was performed to exclude systemic autoimmune disease, primary CNS vasculitis, and rapidly progressive neurodegenerative disorders. A broad serum autoimmune screen was negative: antinuclear, anti-Ro, anti-La, anti-Sm, and anti-RNP antibodies (against systemic lupus erythematosus, Sjögren syndrome, and connective-tissue disease), antineutrophil cytoplasmic antibodies (against ANCA-associated vasculitis), and antiphospholipid antibodies (against antiphospholipid syndrome). These negative results argued against a systemic autoimmune or vasculitic disorder as the cause of the presentation, supporting a primary CNS autoimmune process. CSF showed no oligoclonal bands or intrathecal IgG synthesis; 14-3-3 protein was negative, and phosphorylated tau-217 was normal, making prion or neurodegenerative encephalopathies unlikely.

The comprehensive neuronal and paraneoplastic antibody panel was negative in CSF, covering cell-surface/synaptic antigens (including NMDA-receptor, LGI1, CASPR2, GABA-B-receptor, AMPA-receptor and DPPX) and intracellular onconeural antigens (including anti-Hu/ANNA-1, anti-Yo/PCA-1, anti-Ri/ANNA-2, CRMP-5 and amphiphysin); these results argued against other antibody-mediated autoimmune encephalitides and paraneoplastic neurological syndromes. CSF cytochemistry showed isolated protein elevation (159 mg/dl) with normal cell count and glucose parameters, suggesting blood-brain barrier dysfunction without pleocytosis, a pattern described in intracellular antigen-mediated encephalitides. 

Neoplastic and Paraneoplastic Screening.

Given the aseptic inflammatory profile of the CSF and the absence of abnormalities on brain MRI, whole-body 18F-fluorodeoxyglucose (18F-FDG PET/CT) was performed to evaluate for a metabolic pattern suggestive of autoimmune encephalitis and to exclude an underlying paraneoplastic etiology. Brain FDG-PET/CT demonstrated diffuse cortical hypermetabolism, a finding compatible with autoimmune encephalitis but also potentially influenced by ongoing or recent ictal activity in prolonged status epilepticus (Figure 1B). No hypermetabolic foci were identified in the thorax, abdomen, pelvis, breast, lymph nodes, or skeletal system, effectively excluding occult systemic malignancy as a paraneoplastic trigger.

Repeat Neuroimaging.

A second brain MRI was performed in the ICU after prolonged status epilepticus. DWI showed frontal cortical restricted diffusion with low ADC values, consistent with cytotoxic edema (Figure 1C). T2-weighted and FLAIR sequences showed frontoparietotemporal cortical and subcortical hyperintensities with preserved ADC values, consistent with vasogenic edema (Figure 1D). The nonvascular distribution argued against embolic or large-vessel ischemia and favored peri-ictal changes from sustained epileptic activity. Bilateral occipital sulcal FLAIR hyperintensity was also present, without leptomeningeal enhancement or mass effect. No new hemorrhagic lesions, abscess, or classic mesial temporal hyperintensity suggestive of limbic encephalitis were identified.

The diagnosis was established based on the autoimmune encephalitis criteria proposed by Graus et al. [9], under which a compatible clinical presentation and the reasonable exclusion of alternative causes support a diagnosis of possible autoimmune encephalitis even when initial antibody testing was negative.

Therapeutic interventions

Empiric Antimicrobial Therapy

Given the febrile encephalopathic presentation and inability to immediately exclude CNS infection, empiric broad-spectrum antimicrobial therapy was started on ICU admission, including intravenous ceftriaxone and vancomycin for bacterial meningitis, acyclovir for herpes simplex encephalitis, and dexamethasone as adjunctive therapy. All agents were sequentially discontinued after CSF cultures, multiplex meningitis/encephalitis PCR, and targeted serological and molecular tests were negative.

Antiseizure Therapy

**Figure 2:**
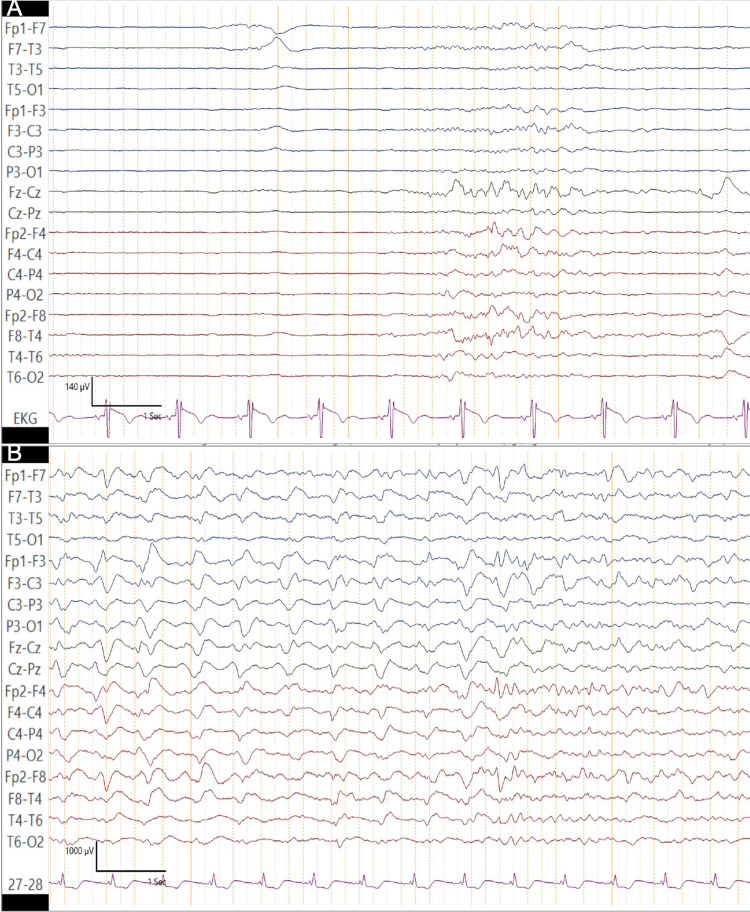
Electroencephalographic findings during treatment of super-refractory status epilepticus (A) Continuous EEG obtained under sedative therapy demonstrated a burst-suppression pattern. (B) Follow-up EEG after withdrawal of sedation showed generalized epileptiform activity characterized by biphasic and triphasic sharp waves, consistent with persistent cortical hyperexcitability EEG: electroencephalography

Seizure control during super-refractory status epilepticus required escalating antiseizure medications. Intravenous levetiracetam was followed by phenytoin, lacosamide, and clonazepam in an attempt to achieve EEG control (Figure 2A). Phenytoin was discontinued early because of Stevens-Johnson syndrome and was replaced with intravenous valproate. Because electrographic seizures persisted for more than 24 hours after the initiation of continuous intravenous midazolam, titrated to burst suppression on video-EEG monitoring, the patient met the accepted definition of super-refractory status epilepticus [8]. Midazolam was subsequently tapered gradually over the following weeks as electrographic seizures resolved (Figure 2B).

Immunotherapy

Although neuronal antibody results were not yet available, the convergence of new-onset super-refractory status epilepticus with encephalopathic features, negative infectious workup, isolated CSF protein elevation without pleocytosis, and diffuse cortical hypermetabolism on 18F-FDG PET/CT supported probable autoimmune status epilepticus. Empiric first-line immunotherapy was therefore started without waiting for CSF antibody confirmation. On ICU day 4, intravenous methylprednisolone 1 g/day was given for five days, followed by a slow oral prednisone taper. Intravenous immunoglobulin (IVIG) was administered concurrently at 0.4 g/kg per day for five days. Combined corticosteroid and IVIG therapy was chosen because of the severe course and evidence supporting early empiric immunotherapy in suspected autoimmune encephalitis, even without antibody confirmation.

Follow-up and outcomes

ICU Course and Neurological Recovery

EEG seizure activity progressively resolved with combined antiseizure therapy and immunotherapy, with the last ictal pattern recorded on ICU day 17. Continuous video-EEG monitoring subsequently showed gradual background normalization. Over the next two weeks, she regained consciousness with progressive improvement in alertness, orientation, and language.

Hospital Discharge

The patient was discharged on day 43. She was fully alert and oriented, with fluent, grammatically intact speech, no residual aphasia, and no focal motor or sensory deficits. Decannulation was successful without respiratory compromise, and percutaneous gastrostomy was removed without complications. Her modified Rankin Scale (mRS) score was 1, indicating no significant disability despite prolonged critical illness [16].

Outpatient Follow-Up

**Figure 3:**
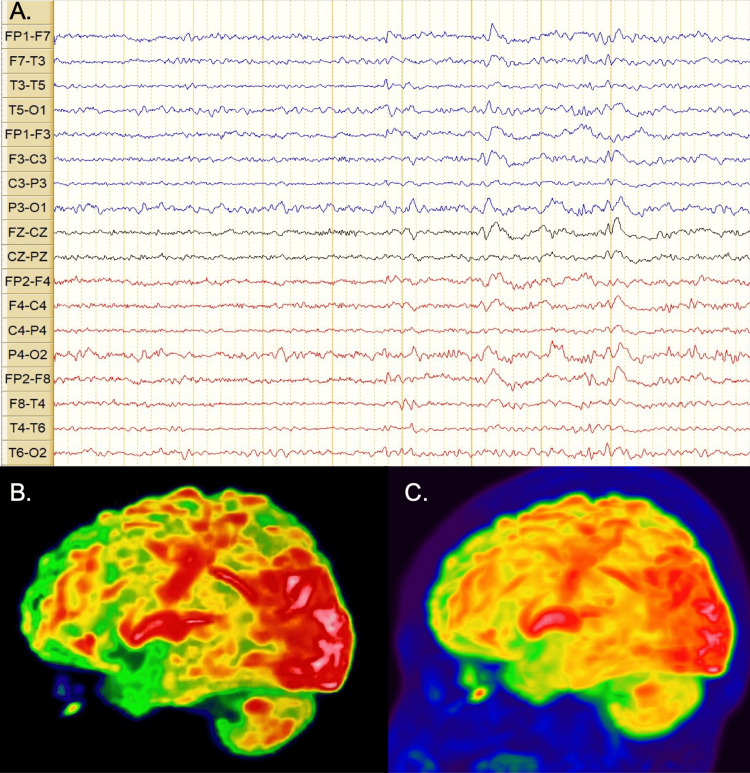
Follow-up cognitive, electroencephalographic, and metabolic assessment after clinical recovery (A) Follow-up EEG obtained during outpatient evaluation showing bilateral frontotemporal focal epileptiform activity, with left-sided predominance. (B) Follow-up brain 18F-FDG PET/CT at one month demonstrating persistent but decreased cortical hypermetabolism (residual high-uptake cortex, red on the color scale, reduced in extent compared with the baseline study in Figure 1B). (C) Follow-up brain 18F-FDG PET/CT at eight months showing further metabolic improvement, with marked reduction of the red (high-uptake) cortical areas EEG: electroencephalography; 18F-FDG PET/CT: 18F-fluorodeoxyglucose positron emission tomography/computed tomography

At neurology follow-up, the patient remained independent in daily living activities, with no motor sequelae or recurrent seizures. However, she reported persistent headaches, insomnia, and restlessness, suggestive of residual encephalitic activity. Montreal Cognitive Assessment (MoCA) score was 26/30 [17]. Of note, 1 point was lost on the trail-making task, 1 point on cube copying, and 2 points in attention after failing to complete the forward and backward digit span tasks. The remaining items were completed correctly. Follow-up continuous EEG showed persistent interictal epileptiform activity despite resolution of cortical edema on brain MRI, suggesting ongoing low-grade autoimmune CNS involvement (Figure 3A). Serum neuronal antibody testing returned positive for anti-GAD65 antibodies at a lower titer of 0.04 nmol/L (reference range: 0.00-0.02 nmol/L), while the remaining neuronal antibodies were negative. The low antibody concentration was interpreted as reflecting prior immunotherapy and, together with the clinical, electrographic, and metabolic findings, supported a probable diagnosis of GAD65 antibody-associated autoimmune encephalitis. Follow-up 18F-FDG PET/CT at one and eight months after discharge showed progressive metabolic improvement (Figures 3B, 3C). Given persistent epileptiform activity, residual symptoms, and slow metabolic normalization, maintenance rituximab every six months was initiated. Lacosamide 300 mg/day and levetiracetam 3 g/day were continued.

Table 1 summarizes the chronology of the hospitalization, including the key diagnostic and therapeutic decisions.

**Table 1:** Clinical timeline and key diagnostic-therapeutic events ED: emergency department; CT: computed tomography; ABCD2: Age, Blood Pressure, Clinical Features, Duration of Symptoms, and Diabetes score; TIA: transient ischemic attack; NIHSS: National Institutes of Health Stroke Scale; MRI: magnetic resonance imaging; DWI: diffusion-weighted imaging; EEG: electroencephalography; ICU: intensive care unit; ¹⁸F-FDG: ¹⁸F-fluorodeoxyglucose; PET/CT: positron emission tomography/computed tomography; HSV: herpes simplex virus; PCR: polymerase chain reaction; HTLV: human T-cell lymphotropic virus; CSF: cerebrospinal fluid; ADA: adenosine deaminase; ANA: antinuclear antibodies; ANCA: antineutrophil cytoplasmic antibodies; SM/RNP: Smith/ribonucleoprotein antibodies; IVIG: intravenous immunoglobulin; C3: complement component 3; C4: complement component 4; anti-GAD65: anti-glutamic acid decarboxylase 65 antibodies; anti-DPPX: anti-dipeptidyl-peptidase-like protein 6 antibodies; anti-GABA-B-R: anti-gamma-aminobutyric acid type B receptor antibodies; anti-GFAP: anti-glial fibrillary acidic protein antibodies; anti-mGluR1: anti-metabotropic glutamate receptor 1 antibodies; anti-IgLON5: anti-IgLON family member 5 antibodies; FOUR: Full Outline of UnResponsiveness; mRS: modified Rankin Scale; MoCA: Montreal Cognitive Assessment

Date	Clinical events and decisions
June 11, 2025	ED visit 1: 20-minute episode of right hemiparesis, dysarthria, and hemifacial palsy, with spontaneous resolution. Brain CT was negative. ABCD2 score: 4. The patient was discharged with a diagnosis of TIA and started on dual antiplatelet therapy
June 19, 2025 (AM)	ED visit 2: tremor, dysarthria, left facial palsy, and hemiplegic gait lasting 20 minutes. NIHSS 2. MRI showed no DWI restriction and Fazekas-1 white matter hyperintensities. EEG showed diffuse encephalopathy with frontotemporal sharp waves, raising suspicion for encephalitis
June 19, 2025 (PM)	Status epilepticus occurred during lumbar puncture, with two tonic-clonic seizures. The patient was intubated and admitted to the ICU. Empiric ceftriaxone, acyclovir, vancomycin, and dexamethasone were started. Levetiracetam, phenytoin, and lacosamide were initiated. Vasopressors were required. EEG showed a burst-suppression pattern
June 21, 2025	^18^F-FDG PET/CT showed diffuse cortical hypermetabolism, an incidental left frontal extra-axial lesion consistent with meningioma, and no paraneoplastic focus. Full infectious workup, including CSF cultures, HSV PCR, Biofire, HTLV, ADA, West Nile, Borrelia, Rickettsia, and enterovirus testing, was negative
June 23, 2025	Empiric methylprednisolone 1 g/day was started for 5 days. Expanded testing, including ANA, ANCA, antiphospholipid, oligoclonal bands, 14-3-3, tau-217, anti SM/RNP, was negative
June 29-July 2, 2025	IVIG was administered for 5 days. Clonazepam was added. Complement levels were mildly reduced: C3 66.2 mg/dL and C4 11.3 mg/dl. The rheumatoid factor was normal
July 1, 2025	Tracheostomy and percutaneous gastrostomy were performed. Tracheal aspirate grew Enterobacter cloacae without resistance genes; nebulized amikacin was administered. CSF autoimmune encephalitis panel returned negative for anti-GAD65, anti-DPPX, anti-GABA-B-R, anti-GFAP, anti-mGluR1, and anti-IgLON5 antibodies
July 5-12, 2025	Midazolam and clonazepam were weaned. Phenytoin was discontinued because of Stevens-Johnson syndrome, and valproate was added
July 8, 2025	Repeat MRI showed frontal cytotoxic cortical edema and frontoparietotemporal vasogenic edema, consistent with a post-ictal pattern. Bilateral occipital sulcal hyperintensity was also present
July 16, 2025	EEG showed only cortico-subcortical dysfunction, without epileptiform activity. FOUR score: 16/16
July 26, 2025	Hyperactive delirium developed. Decannulation was performed without respiratory compromise, and the stoma closed subsequently
August 1, 2025	Discharge, hospital day 43: the patient had no significant disability (mRS 1), tolerated an oral diet, had spontaneous micturition, and had undergone gastrostomy removal
August 28, 2025	Outpatient neurology follow-up. ^18^F-FDG PET/CT showed interval metabolic improvement compared with the baseline study. Persistent interictal epileptiform activity on EEG despite radiological resolution of cortical edema. Residual symptoms reported (headaches, insomnia, and restlessness). MoCA 26/30
Post-discharge	Serum autoimmune encephalitis testing was positive for anti-GAD65 at 0.04 nmol/L (reference range 0.00-0.02 nmol/L). All other antibodies were negative, including anti-DPPX, GABA-B-R, GFAP, mGlurR1, and IgLON5. Diagnosis of anti-GAD65 autoimmune encephalitis supported
September 2025	Maintenance immunotherapy with rituximab initiated, scheduled every 6 months, based on persistent interictal epileptiform activity, residual symptoms, and slow normalization of brain metabolism on follow-up PET. Maintenance antiseizure therapy with lacosamide 300 mg/day and levetiracetam 3 g/day continued
March 5, 2026	^18^F-FDG PET/CT follow-up with metabolic improvement and marked reduction of cortical hypermetabolism

Table 2 presents a summary of the key laboratory findings from the diagnostic workup.

**Table 2:** Key laboratory readings during the diagnostic workup AFB: acid-fast bacilli; ANCA: antineutrophil cytoplasmic antibodies; aPTT: activated partial thromboplastin time; CMV: cytomegalovirus; CNS: central nervous system; CSF: cerebrospinal fluid; CU: chemiluminescence units; dRVVT: dilute Russell viper venom time; eGFR: estimated glomerular filtration rate; GAD65: glutamic acid decarboxylase 65; HDL/LDL: high-/low-density lipoprotein; HHV-6: human herpesvirus 6; HSV: herpes simplex virus; IFA: indirect immunofluorescence assay; INR: international normalized ratio; RNP: ribonucleoprotein; VZV: varicella-zoster virus

Test	Result	Reference range	Interpretation
A. Admission blood work and vascular-risk profile			
Hemoglobin	15.7 g/dL	12–16 g/dL	Normal
Platelets	263 ×10⁹/L	150–450 ×10⁹/L	Normal
Leukocytes (blood)	4.47 ×10⁹/L	4.0–11.0 ×10⁹/L	Normal
Glucose	110 mg/dL	70–100 mg/dL	Slightly elevated
Creatinine (eGFR)	0.72 mg/dL (86 mL/min/1.73m²)	0.6–1.1 mg/dL	Normal renal function
Sodium/potassium	133 / 5.0 mmol/L	135–145/3.5–5.1	Na mildly low
Total cholesterol	235 mg/dL	< 200 mg/dL	Elevated
LDL cholesterol	154 mg/dL	< 100 mg/dL	Elevated
HDL cholesterol	59 mg/dL	> 50 mg/dL (women)	Normal
Triglycerides	112 mg/dL	< 150 mg/dL	Normal
C-reactive protein	0.6 mg/dL	< 0.5 mg/dL	Minimally elevated
Procalcitonin	0.03 ng/mL	< 0.10 ng/mL	Low - against bacterial infection
INR/aPTT	0.98/24.3 s	0.8–1.2/25–35 s	Normal coagulation
B. Cerebrospinal fluid cytochemistry and cell count			
Appearance/color	Transparent/colorless	Transparent/colorless	Normal
Leukocytes	2 cells/µL	0–10 cells/µL	Normal
Erythrocytes	0 cells/µL	0–10 cells/µL	Non-traumatic tap
Total protein	159 mg/dL	15–45 mg/dL	Elevated
Glucose	65 mg/dL	40–70 mg/dL	Normal
Oligoclonal bands^*^	Absent	Absent	Negative
14-3-3 protein^*^	Negative	Negative	Prion disease unlikely
Phosphorylated tau-217^*^	Normal	—	Neurodegeneration unlikely
Adenosine deaminase^*^	Normal	—	Against tuberculous infection
C. CSF infectious workup			
Gram stain; bacterial culture (7 d)	No organisms; no growth	No growth	Negative
FilmArray meningitis/encephalitis multiplex PCR (E. coli K1, H. influenzae, L. monocytogenes, N. meningitidis, S. agalactiae, S. pneumoniae, CMV, enterovirus, HSV-1, HSV-2, HHV-6, human parechovirus, VZV, C. neoformans/gattii)	All not detected	Not detected	No CNS pathogen
India ink/cryptococcal	Negative	Negative	No Cryptococcus
KOH/fungal culture (4 wk)	No elements; no growth	No growth	Negative
AFB smear; mycobacterial culture (8 wk)	Negative; no growth	No growth	No mycobacteria
Anaerobic culture	No growth	No growth	Negative
D. CSF autoimmune encephalitis antibody panel (Mayo Clinic Laboratories; radioimmunoassay and IFA)			
Anti-GAD65 (CSF)	0.00 nmol/L	0.00–0.02 nmol/L	Negative
Remaining 23 antibodies: AGNA-1, AMPA-R, amphiphysin, ANNA-1/2/3, CASPR2, CRMP-5, DPPX, GABA-B-R, GFAP, mGluR1, IgLON5, LGI1, neurochondrin, NIF, NMDA-R, PCA-1/2/Tr, PDE10A, septin-7, TRIM46	All negative	Negative	No CSF antibody detected
E. Serum systemic autoimmune/antiphospholipid screen			
Antinuclear antibodies (IFA)	Negative; titer < 1:40	< 1:40	Negative
C-ANCA/p-ANCA	Negative	Negative	No vasculitis
Lupus anticoagulant (aPTT 38 s; dRVVT 35 s)	Not detected	Not detected	Negative
Anticardiolipin IgG/IgM/IgA	2.7/1.3/2.2 CU	< 20 CU	Negative
Beta-2 glycoprotein I IgG/IgM/IgA	8.1/<1.1/< 4.0 CU	< 20 CU	No antiphospholipid syndrome
Anti-Ro/SS-A/anti-La/SS-B	<4.9/<3.3 CU	< 20 CU	Negative
Anti-Sm/anti-RNP	Negative	Negative	Negative
Rheumatoid factor	12 IU/mL	< 14 IU/mL	Negative
Complement C3	66 mg/dL	79–152 mg/dL	Low
Complement C4	11 mg/dL	16–38 mg/dL	Low
F. Serum neuronal antibody panel(Mayo Clinic Laboratories; radioimmunoassay and IFA; post-immunotherapy)			
Anti-GAD65 (serum)	0.04 nmol/L	0.00–0.02 nmol/L	Positive by cutoff; low titer
Remaining 23 antibodies (same panel as D)	All negative	Negative	No other neuronal antibody
G. Other infectious serologies			
HTLV-1/HTLV-2	Negative	Negative	—
West Nile virus IgM	Negative	Negative	—
Measles; enterovirus (serum)	Negative	Negative	—
Rubella IgG/IgM	18.6 (positive)/< 10 (negative)	IgG > 10 = immune	Past immunity; no acute infection
Borrelia burgdorferi IgG/IgM/total	Negative	Negative	No Lyme disease
Rickettsia panel	Negative	Negative	—
HIV; hepatitis profile	Negative	Negative	—

## Discussion

This case highlights three clinically important features that distinguish it from typical anti-GAD65 encephalitis: recurrent transient focal neurological episodes initially misattributed to TIA; rapid progression to super-refractory status epilepticus requiring a 43-day ICU stay; and negative initial CSF autoimmune testing, with later low-titer serum positivity supporting the diagnosis during follow-up.

Anti-GAD65 antibodies impair GABAergic neurotransmission by reducing synaptic GABA availability, lowering the threshold for focal cortical hyperexcitability and potentially causing transient ictal events that mimic TIA [1,2,7]. Mild carotid atheromatous disease and Fazekas-1 white matter changes provided a plausible vascular alternative, increasing the diagnostic challenge [15]. In retrospect, the initial transient episodes are best interpreted as focal ictal phenomena, such as ictal aphasia and focal motor seizures, arising from cortical hyperexcitability rather than as ischaemic events. Nevertheless, the initial diagnosis of TIA was reasonable, as the first episode was focal, transient, and self-limited, with an unremarkable vascular workup and risk stratification based on the validated ABCD2 score. Thus, this case does not suggest that the initial evaluation was erroneous; rather, it illustrates the importance of reconsidering an autoimmune mimic as the clinical course evolves.

The day-19 cytotoxic and vasogenic MRI pattern was consistent with prolonged epileptic encephalopathy [18]. Although anti-GAD65 encephalitis typically presents as limbic encephalitis, cases dominated by super-refractory status epilepticus have been reported only in small series [3,5,19]. In these predominantly retrospective cohorts, the diagnosis rested on GAD65 seropositivity (typically at high titer) with a compatible limbic or epileptic phenotype and exclusion of alternative causes; management combined multiple antiseizure medications with first-line immunotherapy (corticosteroids, IVIG, and/or plasma exchange), frequently escalating to second-line rituximab because GAD65-associated epilepsy is often only partially responsive to immunotherapy, and reported outcomes were variable, with many patients retaining chronic epilepsy [3,5,19].

The negative initial CSF result may reflect antibody concentrations below the assay detection threshold or limitations of single-compartment testing. Anti-GAD65 syndromes show considerable heterogeneity in their serum-CSF distribution, with intrathecal synthesis documented in some phenotypes, such as stiff person syndrome [20]. Because anti-GAD65 targets an intracellular epitope and disease pathogenesis is thought to be predominantly mediated by cytotoxic CD8+ T lymphocytes rather than direct antibody-mediated neuronal injury [21], antibody titers may not parallel disease activity and may be affected by recent immunotherapy.

Unlike surface-antigen encephalitides, in which serum positivity more often parallels CSF findings [6], a negative CSF panel does not exclude anti-GAD65 encephalitis, and dual serum-CSF testing may be required [9,22]. In this case, discordance followed an inverse, less commonly described pattern: the acute-phase CSF autoimmune panel was negative, whereas serum testing one month after discharge, after immunotherapy had sustained clinical recovery, was positive. However, this low serum titer could understate the true antibody burden because of prior immunotherapy. Conversely, low-titer serum GAD65 positivity is not fully specific and can occur in type 1 diabetes and other organ-specific autoimmunity; higher-specificity confirmation (paired or repeat serum), CSF testing, or demonstration of intrathecal synthesis was of limited additional yield here, given the low, treatment-confounded titre.

Treatment of anti-GAD65 encephalitis lacks randomized trial evidence. First-line immunotherapy (corticosteroids, IVIG, plasma exchange) is recommended empirically, whereas second-line agents (rituximab and mycophenolate) are reserved for refractory or relapsing disease [23], in line with best-practice recommendations for autoimmune encephalitis [9,24] and established treatment protocols for super-refractory status epilepticus [8]. This patient’s favorable outcome, with return to her premorbid baseline despite prolonged ICU course, supports early empiric immunotherapy without awaiting antibody confirmation [3,23,24]. The close temporal link between immunotherapy and clinical-electrographic improvement supports a therapeutic effect, although a single case cannot establish causality with certainty. The substantial resource burden (tracheostomy, gastrostomy, 43-day ICU stay and broad-spectrum antimicrobials) highlights the cost of diagnostic delay in this treatable condition [8].

## Conclusions

Anti-GAD65 autoimmune encephalitis may present with cerebrovascular-like episodes before progressing to super-refractory status epilepticus. In clinically compatible syndromes, a negative initial CSF autoimmune panel should not exclude the diagnosis; concurrent or repeat serum testing, particularly after stabilization or empiric immunotherapy, may provide important supporting evidence. Early empiric immunotherapy based on clinical and paraclinical findings, without awaiting antibody confirmation, was associated with a return to the patient’s premorbid functional baseline despite prolonged critical illness, although residual symptoms and EEG abnormalities required maintenance immunotherapy. This report underscores the importance of integrating clinical, EEG, CSF, and metabolic imaging findings in patients with initially CSF-negative suspected autoimmune encephalitis, in which individually nonspecific findings support a probable rather than definitive diagnosis.

## References

[1] Saiz A, Blanco Y, Sabater L (2008). Spectrum of neurological syndromes associated with glutamic acid decarboxylase antibodies: diagnostic clues for this association. Brain.

[2] Vianello M, Tavolato B, Giometto B (2002). Glutamic acid decarboxylase autoantibodies and neurological disorders. Neurol Sci.

[3] Malter MP, Helmstaedter C, Urbach H, Vincent A, Bien CG (2010). Antibodies to glutamic acid decarboxylase define a form of limbic encephalitis. Ann Neurol.

[4] Honnorat J, Saiz A, Giometto B (2001). Cerebellar ataxia with anti-glutamic acid decarboxylase antibodies: study of 14 patients. Arch Neurol.

[5] Budhram A, Sechi E, Flanagan EP (2021). Clinical spectrum of high-titre GAD65 antibodies. J Neurol Neurosurg Psychiatry.

[6] Dalmau J, Graus F (2018). Antibody-mediated encephalitis. N Engl J Med.

[7] Muñiz-Castrillo S, Vogrig A, Joubert B (2020). Transient neurological symptoms preceding cerebellar ataxia with glutamic acid decarboxylase antibodies. Cerebellum.

[8] Shorvon S, Ferlisi M (2011). The treatment of super-refractory status epilepticus: a critical review of available therapies and a clinical treatment protocol. Brain.

[9] Graus F, Titulaer MJ, Balu R (2016). A clinical approach to diagnosis of autoimmune encephalitis. Lancet Neurol.

[10] Johnston SC, Rothwell PM, Nguyen-Huynh MN, Giles MF, Elkins JS, Bernstein AL, Sidney S (2007). Validation and refinement of scores to predict very early stroke risk after transient ischaemic attack. Lancet.

[11] Wang Y, Wang Y, Zhao X (2013). Clopidogrel with aspirin in acute minor stroke or transient ischemic attack. N Engl J Med.

[12] Johnston SC, Easton JD, Farrant M (2018). Clopidogrel and aspirin in acute ischemic stroke and high-risk TIA. N Engl J Med.

[13] Kleindorfer DO, Towfighi A, Chaturvedi S (2021). 2021 guideline for the prevention of stroke in patients with stroke and transient ischemic attack: a guideline from the American Heart Association/American Stroke Association. Stroke.

[14] Lyden P, Brott T, Tilley B (1994). Improved reliability of the NIH Stroke Scale using video training. NINDS TPA Stroke Study Group. Stroke.

[15] Fazekas F, Chawluk JB, Alavi A, Hurtig HI, Zimmerman RA (1987). MR signal abnormalities at 1.5 T in Alzheimer's dementia and normal aging. AJR Am J Roentgenol.

[16] van Swieten JC, Koudstaal PJ, Visser MC, Schouten HJ, van Gijn J (1988). Interobserver agreement for the assessment of handicap in stroke patients. Stroke.

[17] Nasreddine ZS, Phillips NA, Bédirian V (2005). The Montreal Cognitive Assessment, MoCA: a brief screening tool for mild cognitive impairment. J Am Geriatr Soc.

[18] Giovannini G, Kuchukhidze G, McCoy MR, Meletti S, Trinka E (2018). Neuroimaging alterations related to status epilepticus in an adult population: definition of MRI findings and clinical-EEG correlation. Epilepsia.

[19] Gastaldi M, Mariotto S, Giannoccaro MP (2020). Subgroup comparison according to clinical phenotype and serostatus in autoimmune encephalitis: a multicenter retrospective study. Eur J Neurol.

[20] Dalakas MC, Li M, Fujii M, Jacobowitz DM (2001). Stiff person syndrome: quantification, specificity, and intrathecal synthesis of GAD65 antibodies. Neurology.

[21] Tröscher AR, Mair KM, Verdú de Juan L (2023). Temporal lobe epilepsy with GAD antibodies: neurons killed by T cells not by complement membrane attack complex. Brain.

[22] Gresa-Arribas N, Ariño H, Martínez-Hernández E (2015). Antibodies to inhibitory synaptic proteins in neurological syndromes associated with glutamic acid decarboxylase autoimmunity. PLoS One.

[23] Quek AM, Britton JW, McKeon A (2012). Autoimmune epilepsy: clinical characteristics and response to immunotherapy. Arch Neurol.

[24] Abboud H, Probasco JC, Irani S (2021). Autoimmune encephalitis: proposed best practice recommendations for diagnosis and acute management. J Neurol Neurosurg Psychiatry.

